# Improving HIV coreceptor usage prediction in the clinic using hints from next-generation sequencing data

**DOI:** 10.1093/bioinformatics/bts373

**Published:** 2012-09-03

**Authors:** Nico Pfeifer, Thomas Lengauer

**Affiliations:** Department of Computational Biology and Applied Algorithmics, Max Planck Institute for Informatics, Campus E1 4, 66123 Saarbrücken, Germany

## Abstract

**Motivation:** Due to the high mutation rate of human immunodeficiency virus (HIV), drug-resistant-variants emerge frequently. Therefore, researchers are constantly searching for new ways to attack the virus. One new class of anti-HIV drugs is the class of coreceptor antagonists that block cell entry by occupying a coreceptor on CD4 cells. This type of drug just has an effect on the subset of HIVs that use the inhibited coreceptor. A good prediction of whether the viral population inside a patient is susceptible to the treatment is hence very important for therapy decisions and pre-requisite to administering the respective drug. The first prediction models were based on data from Sanger sequencing of the V3 loop of HIV. Recently, a method based on next-generation sequencing (NGS) data was introduced that predicts labels for each read separately and decides on the patient label through a percentage threshold for the resistant viral minority.

**Results:** We model the prediction problem on the patient level taking the information of all reads from NGS data jointly into account. This enables us to improve prediction performance for NGS data, but we can also use the trained model to improve predictions based on Sanger sequencing data. Therefore, also laboratories without NGS capabilities can benefit from the improvements. Furthermore, we show which amino acids at which position are important for prediction success, giving clues on how the interaction mechanism between the V3 loop and the particular coreceptors might be influenced.

**Availability:** A webserver is available at http://coreceptor.bioinf.mpi-inf.mpg.de.

**Contact:** nico.pfeifer@mpi-inf.mpg.de

## 1 INTRODUCTION

Since the discovery of the human immunodeficiency virus (HIV) in 1983 (Barr-Sinoussi *et al.*, 1983; Gallo *et al.*, 1984) there has been an enormous attention of researchers to HIV and the associated disease, the acquired immune deficiency syndrome. The first drug against HIV was already introduced in 1985 (Mitsuya *et al.*, 1985), but it soon became obvious that additional anti-HIV drugs would be needed due to the high mutation rate of the virus. Even with the introduction of combination drug therapy in the mid 1990s, the viral populations of HIV-infected patients under therapy could become resistant after a certain period of time-creating the need for further anti-HIV drugs that attack the virus in a different manner. Therefore, researchers are constantly searching for new ways to limit viral load in HIV-infected patients. HIV needs a coreceptor on CD4 cells to enter the cell (Lusso, 2006). Different HIV strains use different coreceptors. The two most prominent coreceptors are CCR5 and CXCR4. The first approved drug that blocks cell entry of CCR5-using viruses (R5 viruses) by serving as a CCR5 antagonist is called maraviroc (MacArthur and Novak, 2008), but there is no approved drug that blocks cell entry of CXCR4-using HIVs (X4 viruses). Administering CCR5 blockers requires use of a companion diagnostic determining viral coreceptor usage, also called viral tropism. For this purpose, Whitcomb *et al.* (2007) introduced a laboratory test called Trofile, which was replaced by the Enhanced Sensitivity Trofile Assay (ESTA) (Reeves *et al.*, 2008), but these methods have a long turnaround time, are costly, are not widely accessible and require large sample volume (Rose *et al.*, 2009). Recently, approaches that predict viral tropism based on the genetic sequence of the V3 loop, which is part of the envelope (Env) protein of HIV, have been introduced (Jensen *et al.*, 2003; Sing *et al.*, 2007). All of these models were trained with either clonal-sequenced data or bulk sequenced data. In the former case, the sequences of individual clones of R5 or X4 viruses are used and in the latter case each sequence represents a consensus of the dominating strains in the whole viral population inside a patient. Such bulk sequences also comprise ambiguous sequence positions, called wobbles. Prediction models based on bulk sequenced data perform in general worse than methods on clonal sequences (Lengauer *et al.*, 2007). Presumably, this is due partly to the fact that bulk consensus sequences describe the dominating strains in a viral population only incompletely and partly because small viral minorities do not show up in bulk data at all. Further improvements were achieved by utilizing other parts of the *env* sequence and information on the three-dimensional structure of the V3 loop of the viral surface gene (Dybowski *et al.*, 2010; Sander *et al.*, 2007). Very recently, prediction models for deep sequencing data of the V3 loop were introduced by Swenson *et al.* (2011). They used next-generation sequencing (NGS) data from the Maraviroc versus Optimized Therapy in Viremic Antiretroviral Treatment-Experienced Patients (MOTIVATE) studies (Fätkenheuer *et al.*, 2008; Gulick *et al.*, 2008) to predict virologic response to maraviroc. This dataset comprises ~3000 reads per patient sample, where each read corresponds to a particular V3 loop of an HIV variant inside the patient. Swenson *et al.* classified each read with standard tools and then classified the whole sample depending on how large the fraction of reads with predicted X4-capable label was. This means that they had to use one cutoff for the method that predicted the label for each read and another cutoff to specify the minimal fraction of X4-capable reads such that the sample was classified as X4-capable. Unfortunately, the authors trained these thresholds directly on 75% of the data that they then used for validation, which is why it is unclear how well the method performs on unseen data. Instead of classifying each read separately, we consider the reads of a sample jointly and train a classifier on this joint representation. This is motivated by the fact that a mere percentage threshold might not have the adequate information for deciding whether a viral population withstands treatment with maraviroc.

Here, we present a method that analyzes the NGS data in a more elaborate fashion. We show that the new method performs better than existing methods without training any parameters on the test data. Furthermore, we introduce new models for predictions based on bulk Sanger sequences and show how to improve predictions with a model trained on NGS data. This is particularly important since many clinics will not have access to NGS techniques for some time to come. Additionally, we show how one can obtain interpretable prediction results and evaluate information on which of the residues of the V3 loop contribute to the improvement of prediction accuracy. Specifically, we find amino acids at certain positions that are highly predictive and might lead to new insights about the interaction between the V3 loop and the different coreceptors.

## 2 METHODS

### 2.1 Data

We analyzed V3 loop sequence data from the MOTIVATE trial (Fätkenheuer *et al.*, 2008; Gulick *et al.*, 2008) and from study A4001029, which were described in detail by Swenson *et al.* (2011). We also had bulk sequenced Sanger sequences from the same patient group. The NGS data were filtered according to the steps described in Swenson *et al.* (2011). This means that we excluded truncated reads that missed four or more bases on either end of the V3 loop. Samples with fewer than 750 reads were removed from the dataset. This resulted in a dataset containing 876 patients with NGS data and bulk sequencing data. For each patient, we had plasma viral load (pVL) measurements at several time points, measured as number of copies per milliliter. For our analysis, we utilized the pVL measurements at baseline, 8 weeks after treatment start and 48 weeks after treatment start. All DNA sequences were translated to amino acid sequences. Then, we created a multiple sequence alignment (MSA) from the Sanger sequences as well as an MSA from the NGS sequences using MUSCLE (Edgar, 2004). Afterwards, we created a joint MSA of all sequences with MUSCLE. We used standard parameters during all MUSCLE runs. The consensus sequence of the final MSA was CTRPNNNTRKSIHIGPGRAFYATGDIIGDIRQAHC (excluding all MSA positions with <1% amino acids). This sequence was recently isolated from an HIV-1-infected patient (Fernández-García *et al.*, 2009) and determined to have the R5 phenotype.

### 2.2 Finding the most representative sequences

The median number of different amino acid sequences for each NGS sample was 47. Since it can be assumed that not all sequences are necessary to predict whether a patient will respond to maraviroc or not, we tried to find a small set of sequences for each patient that best represents the diversity of all present viral sequences. This problem is similar to determining the population structure in a sample. One of the most recent approaches for determining these different subpopulations utilizes a Dirichlet process mixture (Zagordi *et al.*, 2011). Applying the tool to our dataset with standard parameters, this approach classified almost all reads as representatives of different subpopulations which is why we used principal component analysis (PCA) instead (Pearson, 1901). Applying PCA to detect hidden population structure has recently been done by Price *et al.* who effectively applied PCA to single-nucleotide polymorphism data to remove the influence of population structure on genome-wide association tests (Price *et al.*, 2006).

**Fig. 1.**
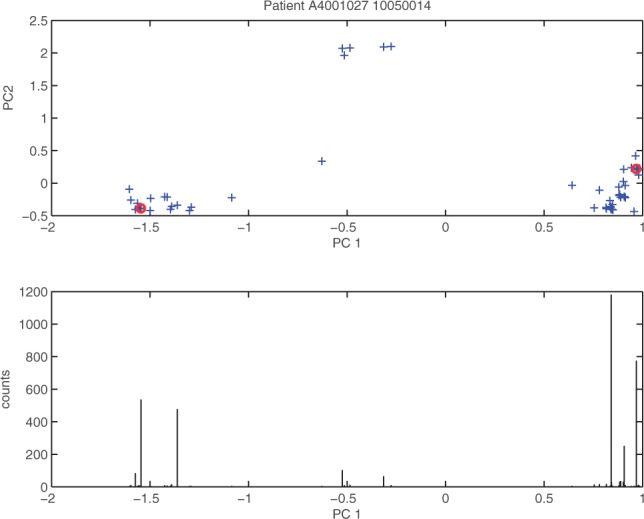
**Choice of representatives**: This plot shows information about the reads of a patient. Each cross in the plot at the top corresponds to a read of the patient and the coordinates are computed by projecting the property encoded reads onto the first and second principal components (PC1 and PC2). The chosen representatives in the PC1 dimension are marked by red circles. The plot at the bottom shows the read counts for the corresponding reads

In order to use PCA, we transformed the amino acid sequences to numerical vectors. We evaluated two different encodings. The first one was a sparse binary encoding where every amino acid is transformed into a bit vector of length 20. Each of these bit vectors has all zeros except for one entry which is at the position of the amino acid in the amino acid alphabet. The final encoding is the concatenation of all of these bit vectors. The second encoding is based on a compact representation of physico-chemical properties of amino acids introduced by Venkatarajan and Braun (2001). Our preliminary analysis showed that the encoding based on physico-chemical properties produced better results which is why we used this encoding in all subsequent experiments. After encoding the sequences, we performed a PCA on the data. Then, we used the first *n* principal components (PCs) that explained 95% of the variance to find good representatives of the variation in the dataset, while *n* was constrained to be smaller than six. For this purpose, we inspected each PC separately and chose one representative from each extreme of the PC. To avoid picking meaningless outliers, we took the read with the highest read count from each boundary region. The boundary region is defined as the smallest region containing the most extreme values on the PC whose reads cumulatively represent at least 5% of the total read counts. An example from one of the patient samples can be seen in Figure 1 for the first PC (PC1). To demonstrate that the information from the different PCs represents different parts of the variation, the plot shows a two-dimensional illustration of the PC space (PC1 and PC2) at the top, but for the choice of the representatives of PC1 only the information represented in the bottom of the figure is important, namely, the read counts and the position according to the actual PC. For each patient sample we also included the two reads with the highest read counts into the set of representatives. Note that it should not hurt the learning algorithm if we include some more representatives than necessary, which is why we chose this conservative approach.

### 2.3 A kernel for NGS data

Previous approaches for predicting coreceptor usage from NGS data were based on considering each read of a patient sample separately (Swenson *et al.*, 2011). Instead of considering the sequences separately, we considered all important reads of a sample jointly. For this purpose, we used the normalized set kernel (NSK) introduced by Gärtner *et al.* (2002) and selected representative sequences using PCA as described above for computing the kernel. Instead of being defined on sequences, the NSK is defined on sets of sequences. Let *χ* be all possible sequences. Then, the NSK for sets *X, X*′ ⊆ *χ* is defined as
(1)
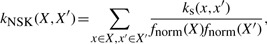

where the inner kernel *k*_s_ is a kernel defined on sequences *x*, *x*′ ∈ *χ* and *f*_norm_(*X*) is a normalization function. Gärtner *et al.* tested different normalization functions. The normalization function that performed well on their dataset was feature space normalization: 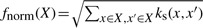 which is why we used this approach in our experiments.

### 2.4 Prediction models for different data scenarios

We built different models to compare performances in different scenarios. Since all models are binary classifiers that predict the coreceptor usage phenotype (X4-capable versus not X4-capable) from the genotype, the name of all models starts with geno2pheno-C. If training and test data are from different sequencing techniques, the training data are named first and the test data are named second. The first scenario is that NGS data are available for all samples. In this case, we extracted the best representatives as described above and used an NSK with a Gaussian radial basis function (RBF) kernel (Schölkopf and Smola, 2001) as the inner kernel function to train a support vector machine (SVM) (Cortes and Vapnik, 1995). This approach is called geno2pheno-C_NGS_. We also tested the NSK based on all reads of a patient instead of just the representatives, but this led to inferior results (data not shown). The second scenario is that only bulk sequenced data from Sanger sequencing are available. In this case we built two different models. The first one was a standard model where each patient was represented by the unique Sanger sequence in which all ambiguous positions were removed. The learning model consisted of an SVM with the Gaussian RBF kernel. This approach is called geno2pheno-C_Sanger_. If there is an ambiguity in the Sanger sequence (e.g. an R meaning that the DNA base is a mixture of A and G), geno2pheno-C_Sanger_ treats this as missing data. This may not be the best way to represent these positions, because they may contain important information on the viral population in a patient. Therefore, we implemented a second approach in which we translated the Sanger sequence of each patient into position-wise probabilities for A, C, G and T. We then translated the sequences into amino acid sequences with corresponding position-wise probabilities. Afterwards, we drew 3000 sequences from each probabilistic representation to obtain a simulated NGS dataset. The mean number of reads for the NGS dataset was 3000 which is why we chose this number. We then used the same methods as for geno2pheno-C_NGS_. This approach is called geno2pheno-C_Sanger^+^_. One of the main goals of this study was to find out if we can improve predictions for bulk sequenced Sanger data with a model trained on NGS data. With the described methods this is now straightforward. One can represent each patient sample either by the unique subsequence as in geno2pheno-C_Sanger_ or by the representatives drawn from the simulated NGS sample as in geno2pheno-C_Sanger^+^_. This can then be used together with the geno2pheno-C_NGS_ model to predict the labels using an NSK and the Gaussian RBF kernel as inner kernel function. The approach with the unique subsequence representation for the test sequences is called geno2pheno-C*_NGS_*_–Sanger_ and the one with the sampled representatives is called geno2pheno-C*_NGS_*_–Sanger^+.^_

### 2.5 Computing significance for predictor comparisons

To evaluate whether an improvement in prediction accuracy between the different prediction models is significant we compared the *log*_10_ pVL reduction at Week 8 for the patient samples classified to be in the *at risk of X4 emergence* group. Therefore, for each two methods in question we tested whether the pVL reduction values of the patients classified to be *at risk of X4 emergence* by the first method and the pVL reduction values of the patients classified to be *at risk of X4 emergence* by the second method came from distributions with different medians using a Wilcoxon rank sum test. Since we knew the direction of the possible median shift, we reported the one-sided *p*-value.

### 2.6 Visualizing the importance of positions inside the V3 loop

After creating a predictive model, it is of interest to evaluate what effect the individual positions in the input sequences have in order to visualize their importance. For linear learning methods such as linear SVMs or linear regression, it is straightforward to determine which positions of the sequence had a certain effect on the prediction. For non-linear SVMs, there have been approaches for string kernels to visualize the importance of k-mers at certain positions by using the support vectors (Meinicke *et al.*, 2004; Sonnenburg *et al.*, 2008). The approach is based on the idea that the differences in the positive and negative support vectors are informative for the prediction because samples more similar to the positive support vectors should be predicted positive and vice versa. This approach is only applicable if there is a finite feature mapping *ϕ*(*x*) such that *ϕ*(*x*_1_) · *ϕ*(*x*_2_) = *k*(*x*_1_,*x*_2_). This is not possible, for example, for the Gaussian RBF kernel for which the samples are mapped into an infinite dimensional feature space (Schölkopf and Smola, 2001). Since we used the Gaussian RBF kernel as an inner kernel function in the NSK, we wanted to generalize the idea. In our approach, we did not use the support vectors directly. Instead, we started with a consensus sample of all samples and changed one letter at a time to determine whether the prediction changed toward the positive or the negative side of the classification boundary. We chose the consensus sequence since it was phenotyped by Fernández-García *et al.* (2009) as R5. Changing one letter at a time is a reasonable approximation since the SVM computes the signed distance from the decision boundary for each test sample *x* by

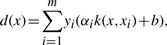

with training samples *x*_1_,*x*_2_,*...,x_m_*, optimized parameters *α* ∈ *R^m^*, offset *b* and class labels *y_i_* ∈ {–1,1}. The training samples *x_i_* with *α_i_ >* 0 are called the support vectors. The distance can be divided into two parts:
(2)
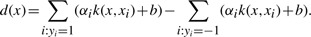

If we change *x*, then *k*(*x,x_i_*) = *ϕ*(*x*)·*ϕ*(*x_i_*) changes, which is the kernel value of *x* and *x_i_*. This kernel value can be considered as a similarity between the two sequences in the feature space that corresponds to the mapping *ϕ*. If the letter change leads to a change toward the negative side of the decision boundary, then either the first term of Equation (2) decreased or the second term increased or both, meaning that the sequence became more similar (has a higher *k*-value) to the negative support vectors compared with the positive support vectors and vice versa for a change toward the positive side. Therefore, one can compute a positional weight of a letter at a certain position by changing the consensus sequence at that position and measuring the change in *d*. More formally, let *s_j_*[*i*] be the letter *c* at position *i* of sequence *s_j_*. We further have the consensus sequence *s*_0_ of length *n* and Σ being the alphabet of all possible letters. Given (letter, position) pairs (*c,i*) with *c* ∈ Σ and 1≤ *i* ≤ *n*, we define the sequence that only differs from the consensus sequence at position *i* with letter *c* as 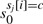. The positional weight of the letter *c* at position *i* is then defined as

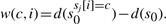

This method is not limited to the kernels we used in our experiments. One can imagine that this strategy also provides interesting insights for an SVM with a graph kernel where one can construct a consensus graph and only change the value of one node at a time to assign weights to the particular nodes according to the difference in prediction. If it is expected that positions are correlated, one can further generalize this idea to change pairs of positions or even higher orders.

### 2.7 Determining the most important positions for prediction success

Given a trained classifier, the training samples and a set of correctly classified sequences evaluating which positions influenced the prediction performance most is an important part of interpreting the model. For this purpose, we compiled a set 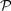 of (position, amino acid) pairs containing all pairs that existed in the set of correctly classified sequences and were different from the (position, amino acid) information of the consensus sequence. Then we constructed two sets of sequences. The first set contained all representatives of all training sequences with a positive class label and the second set contained all representatives of all training sequences with a negative class label. We then performed Fisher's exact test for all elements of 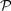 using the positive and the negative set.

In our dataset, we were particularly interested in finding cases where the additional reads from the NGS sample helped to improve predictions. Therefore, we removed each modal sequence, the sequence with the highest read count, for each set of representatives before constructing the positive and the negative set as described above. We then again performed a Fisher's exact test on all elements of 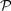. The important (position, amino acid) pairs were the ones that had a smaller *p*-value in the second test and were significant after Bonferroni correction (*p*-value ≤ 0.05/|
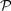
|).

## 3 RESULTS

This section is structured into three main parts. In the first part, we show that by considering the reads of a patient jointly our new method that uses NGS data has higher predictive power than established methods. Furthermore, we can show that one can use our NGS model to improve performance for predictions based on Sanger sequences. This is particularly important since many clinics are still using this sequencing technology. Thus, any improvement directly has an impact. In the second part, we demonstrate how we can use our model to visualize which sequence positions are most informative about the classification. The third part shows that the most significant sequence positions are localized at structurally important places.

### 3.1 Performance comparison

Since we used the same dataset as Swenson *et al.* (2011), we could directly compare the performance of our and their method. They used the NGS data in a different way than we did. Their prediction method consisted of two steps. First, the label of each read was predicted using either geno2pheno (Sing *et al.*, 2007) or WebPSSM (Jensen *et al.*, 2003). In the second step, they classified the sample of a patient as X4-capable if 2% of the reads or more had an X4-capable label. Otherwise, they classified the sample as R5. It has to be noted that they used 75% of the test data to determine the cutoffs of their method (the geno2pheno and WebPSSM cutoffs as well as the cutoff to decide at which percentage of X4-capable reads the sample is classified as X4-capable). In our evaluation we did not train on any test data. Instead, we performed a nested cross-validation (CV) and reported performance on the held-out test partitions. Since every part of the data was in a test partition at one point of the evaluation, we could obtain a test performance on the whole dataset.

**Fig. 2.**
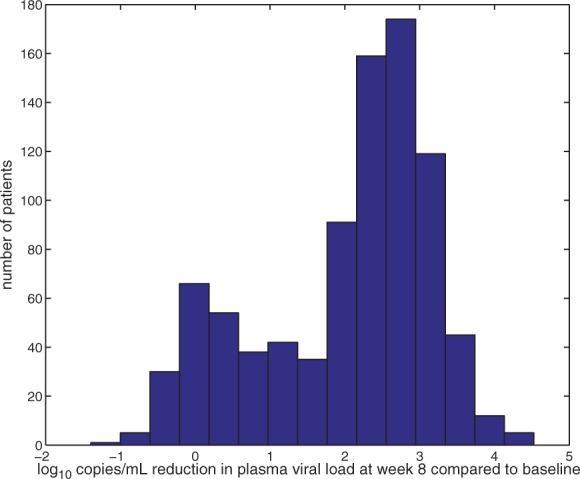
**Fold reduction in plasma viral load (PVL)**: This plot shows the log_10_ reduction in PVL at week eight compared with baseline levels measured as copies/ml

The dataset contained viral load measurements at various different time points. Swenson *et al.* suggested that the pVL measurement at week eight was a good indicator of treatment success, which is why we used it to compute training and test labels. Since we wanted to classify the viral samples of patients according to whether they induced failure of maraviroc treatment, we had to decide which reduction in viral load between baseline and week eight was considered too little. Figure 2 shows that there is a bimodal distribution of the pVL reduction. It seems that for a log_10_ reduction in viral load larger than two it is likely that the patient belongs to the right group (the group with significant pVL reduction). Therefore, we used this value as a threshold, but the choice of this threshold did not vary performance results significantly as long as it was chosen within a reasonable range (log_10_ reduction between 1.5 and 2.1, which corresponds to a reduction by factors between 31 and 126 of the pVLs). In previous coreceptor usage prediction approaches R5 sequences were assigned a negative label and X4-capable sequences obtained a positive label. Therefore, we used *at risk of X4 emergence* as the positive class and *not at risk of X4 emergence* as the negative class instead of treatment failure and treatment success.

To compare performances of the different approaches we ran a 5-fold nested CV on the dataset. This means that we randomly split the dataset into five parts, performed an inner CV on four of the five parts to determine the best parameters and then measured the performance on the left-out fifth part. This was done for all possible combinations. We repeated this process five times to minimize random effects and report the mean performance over these five runs in the following. In order to compare our performances to the performances by Swenson *et al.*, we reported the same measures as they did. One measure they introduced was the pVL reduction of the patients after eight weeks of treatment. If a method only classified patients randomly into the two classes one would expect to obtain the same median pVL reduction for both classes. The more the medians differed because of a higher pVL reduction for the patients in the *not at risk of X4 emergence* class and a lower pVL reduction in the *at risk of X4 emergence* class the better was the method, because it was able to distinguish patients that could benefit from maraviroc treatment from the ones that could not.

The performance comparison of the different approaches is listed in Table 1. The first number in each cell gives the median log_10_ pVL reduction in patients who were classified as *no risk of X4 emergence* (–) and the second number gives the median log_10_ pVL reduction in patients who were classified as *at risk of X4 emergence* (+). As mentioned above, the first number should be as high as possible while the second number is the better the smaller it is for the purpose of clean separation between the two classes. It can be seen that the standard method on Sanger sequences performed badly. We could improve on the performance of this baseline method by incorporating the uncertainties at ambiguous sequence positions (geno2pheno-C_Sanger^+^_). Additionally, we could show that test performance increased if a model trained with NGS data was available, while the test set based on the sampled representatives (geno2pheno-C_NGS–Sanger^+^_) had slightly higher predictive power than the approach that utilized the unique Sanger subsequence (geno2pheno-C_NGS–Sanger_). The improvement from geno2pheno-C_Sanger_ to geno2pheno-C_NGS–Sanger^+^_ was highly significant (*p*-value = 6.22e – 05), while it was only significant at significance level *α* = 0.11 or higher for the improvement from geno2pheno-C_Sanger^+^_ to geno2pheno-C_NGS–Sanger^+^_ (*p*-value = 0.11). As expected the best performance could be achieved if training and test data were NGS data (geno2pheno-C_NGS_). The improvement from geno2pheno-C_NGS–Sanger^+^_ to geno2pheno-C_NGS_ was highly significant (*p*-value = 0.0019). The method had even higher predictive power than the method by Swenson *et al.* since the median pVL reduction was smaller in the (+) group (1.0 compared with 1.4), while it was similar for the (−) group (2.4 for both approaches). Nevertheless, the improvement compared with an implementation of the method by Swenson *et al.* in which we learnt the thresholds in a nested CV was not significant (data not shown). It has to be noted that we did not expect to obtain a value of 0 for the (+) group since the patients were treated not only with maraviroc, but also with a combination of other anti-HIV drugs that were specifically tailored to the HIV viruses in the study participants.

**Table 1.** log_10_ reduction in pVL after eight weeks

	no risk	at risk	Δ
geno2pheno-C_Sanger_	2.4	2.3	0.1
geno2pheno-C_Sanger^+^_	2.4	2.1	0.3
geno2pheno-C_NGS–Sanger_	2.4	1.9	0.5
geno2pheno-C_NGS–Sanger^+^_	2.4	1.7	0.7
geno2pheno-C_NGS_	2.4	1.0	1.4
Swenson *et al.*^a^	(2.4)	(1.4)	(1.0)

Performance comparison for the different methods regarding the median reduction of log_10_ pVL in the different patient groups, measured in copies/ml eight weeks after the beginning of the treatment: The first number corresponds to patients that had been classified as *no risk of X4 emergence* and the second number is for the patients classified as *at risk of X4 emergence*. The third number corresponds to the difference between the medians of the ‘no risk’ and the ‘at risk’ group. The larger the Δ the better the prediction method is able to distinguish patients with treatment success from patients without treatment success.

^a^In contrast to the other approaches, parts of the test data were utilized to optimize model parameters.

**Table 2.** Sustained virologic response

	no risk	at risk
geno2pheno-C_Sanger_	47%	42%
geno2pheno-C_Sanger^+^_	46%	36%
geno2pheno-C_NGS–Sanger_	47%	33%
geno2pheno-C_NGS–Sanger^+^_	47%	31%
geno2pheno-C_NGS_	48%	22%
Swenson *et al.*^a^	(49%)	(26%)

Performance of the different methods regarding sustained virologic response defined as having a pVL lower than 50 copies/ml at week 48: The first number is the percentage of patients with a sustained virologic response that had been classified as *no risk of X4 emergence* and the second number gives this percentage for the patients classified as *at risk of X4 emergence*.

^a^In contrast to the other approaches, parts of the test data were utilized to optimize model parameters.

Another performance measure mentioned in Swenson *et al.* was the proportion of people with a sustained virologic response. This classification was positive for patients having a pVL lower than 50 copies/ml at Week 48. Similar to the reduction in pVL, for a good predictor the percentage of patients with sustained virologic response in the *no risk of X4 emergence* class should be as high as possible, while it should be as low as possible for the people classified *as at risk of X4 emergence*. The performance according to this measure repeated the patterns from the previous measure, meaning that we could improve on test performance for Sanger data by using the model that incorporates positional uncertainties. Furthermore, the model trained on NGS data improved the Sanger data test set predictions, again slightly favoring the approach based on the sampled representatives. Additionally, our model that only uses NGS data for training and test had higher predictive power than the approach by Swenson *et al.*, although the improvement was not statistically significant compared to a nested CV implementation of the method by Swenson *et al.* (data not shown).

### 3.2 Important positions for prediction success

**Fig. 3.**
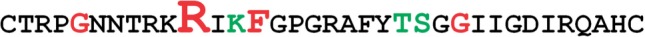
**Visualization example of a prediction**: This plot shows the visualization of the positional weights as described in the Methods section for one test sequence during one of the nested CV runs of geno2pheno-C_NGS–Sanger_. The letters are depicted in different colors according to the positional weights. The red color corresponds to the *at risk of X4 emergence* class. Positions that make the sequence more similar to *no risk of X4 emergence* sequences are marked in green. The higher the positional weight, the larger is the corresponding letter

Interpretation of predictions is a central issue in this field. Very often researchers restrain themselves to simple linear methods for purposes of interpretability and while sacrificing performance. Since we did not use a linear learning algorithm, we showed in Section 2 how to derive meaningful positional weights to obtain interpretable results without sacrificing performance. An example for one of the test sequences in a geno2pheno-C_NGS–Sanger_ run can be seen in Figure 3. For this sequence, the two positions with the highest weights were positions 11 and 14. Specifically, an R at position 11 and an *F* at position 14 were most predictive for the *at risk of X4 emergence* class. One could generalize the idea to the sampled representatives of geno2pheno-C_NGS–Sanger^+^_ by weighting the different letters per position with their occurrences.

**Fig. 4.**
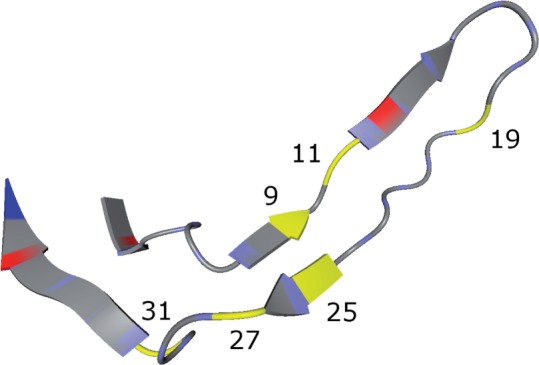
**Bound V3 loop with highlighted important positions**: This plot shows the V3 loop of the PDB structure 2QAD (Huang *et al.*, 2007), visualized with BALLView (Hildebrandt *et al.*, 2010). The important positions are highlighted in yellow

We were interested in the (position, amino acid) pairs that had a lower *p*-value in the set without the modal sequence than in the set with the modal sequence, because this points to extra information provided by the NGS training data. Therefore, we performed a statistical test as described in Section 2. This was done for each of the five runs of the nested CV of geno2pheno-C_NGS–Sanger_, but one could easily adapt this approach to the sampled representatives of geno2pheno-C_NGS–Sanger^+^_. The pairs remaining significant after Bonferroni correction are shown in Table 3. The 11/25 rule is one of the simplest ways to predict coreceptor usage based on the V3 loop sequence (Jensen *et al.*, 2003). In this approach, one checks for basic side chains at position 11 or 25, implicating that these positions are most important for prediction success. Positions 11 and 25 were part of the positions for which we found a significant (position, amino acid) pair. Figure 4 shows the three-dimensional structure of a bound V3 loop with the important positions highlighted in yellow. One can see that position nine is in close proximity to position 25, which supports the hypothesis that position nine is important for the prediction, because one can expect that a change at position 25 affects the binding to the amino acid at position nine and vice versa. Position 19 was also identified as a polymorphic position in information-theoretic analyses by Korber *et al.* (1993). Note that in their analysis the numbering starts at the second letter of the V3 loop which is why position 19 in our analysis corresponds to number 18 in their analysis. Bickel *et al.* (1996) analyzed a larger dataset than Korber *et al.* using the same numbering and, among other covarying positions, they found covariability between position 26 and position 7 (27 and 8 in our analysis). This interaction is also supported by the V3 loop structure in Figure 4. All these hints from the literature support the hypothesis that the amino acid changes at the positions of the significant pairs of our analysis may impact the conformation of the V3 loop in terms of whether it can bind to the CCR5 coreceptor or the CXCR4 coreceptor. We were particularly interested in the changes that improve the prediction and are not supported by the modal sequence, to evaluate which additional benefit can be achieved by using NGS data instead of Sanger sequences. Our approach is able to leverage the information stemming from the more detailed resolution of the viral population afforded by NGS data to infer signals in Sanger sequences that orginate from viral minorities that make their mark on bulk sequences but do so insufficiently for be of benefit to the learning algorithm. The ability of geno2pheno-C_NGS–Sanger_ and geno2pheno-C_NGS–Sanger^+^_ to pick up these signals is one possible explanation for the fact that these methods had higher predictive power than geno2pheno-C_Sanger_ and geno2pheno-C_Sanger^+^_.

**Table 3.** Important positions for prediction success

	(pos, aa): *p*-value	(pos, aa): *p*-value	(pos, aa): *p*-value
run 1	(9,K): 5.88405e–08	(11,R): 1.09165e–06	(27,V): 3.09939e–05
run 2	(9,K): 3.71415e–07	(11,R): 2.24278e–05	(27,V): 2.75498e–05
run 3	(9,K): 1.23451e–05		
run 4	(19,V): 8.1314e–05	(31,K): 1.25565e–05	
run 5	(11,R): 1.32773e–07	(19,V): 1.37038e–05	(25,A): 3.75772e–05

This table shows the (position, amino acid) pairs that were significant after Bonferroni correction and had a smaller *p*-value in the test without the modal sequence representatives.

## 4 CONCLUSION

Due to the high mutation rate of HIV, the viral populations inside a patient are constantly changing. This is one of the main reasons why until today there is no approved vaccine against HIV. It is also the main reason why researchers constantly have to find new ways to attack the virus. One of the new classes of anti-HIV drugs are CCR5 coreceptor antagonists. Since they are only effective against R5 viruses, it is very important to apply a reliable and fast companion diagnostic before administering this type of drug to a patient. Phenotypic tests such as Trofile or ESTA have a long turnaround time, are costly and not easily accessible, and require a large sample volume, which is why an accurate genotypic test has many advantages. We were able to build a superior predictor by prioritizing the most informative strains in a viral population and crafting a more complex information merging procedure based on a set kernel, rather than uniformly processing all strains occurring in the population and merging information by using simple cutoffs. Furthermore, we could show how to construct better predictors for bulk sequenced Sanger sequences. This is very important since few clinics have the means to routinely sequence patient samples with NGS techniques. Additionally, we provided a method to visualize how informative particular positions in the sequences are in terms of the final prediction. We were also able to show which amino acids at which positions were probably responsible for the performance gain. We used this information to speculate about the structural differences between the V3 loops of R5 and X4 viruses and might help to identify important interactions between the V3 loop and the particular coreceptors.

Our framework is very general. One can easily incorporate additional features of interest (e.g. structural descriptors) by using different feature encodings or other kernel functions in the inner kernel. Furthermore, this approach is not limited to predicting coreceptor usage and should be applicable to a wide variety of prediction problems with NGS data, especially in cases where the whole region of interest can be covered by one read.
